# Vitamin D, Bone Metabolism, and Fracture Risk in Polycystic Ovary Syndrome

**DOI:** 10.3390/metabo11020116

**Published:** 2021-02-18

**Authors:** Flavia Di Bari, Antonino Catalano, Federica Bellone, Gabriella Martino, Salvatore Benvenga

**Affiliations:** 1 Department of Clinical and Experimental Medicine, University of Messina, Messina, Viale Gazzi, 98125 Messina, Italy; flaviadb1983@libero.it (F.D.B.); fbellone@unime.it (F.B.); martinog@unime.it (G.M.); s.benvenga@live.it (S.B.); 2 Master Program on Childhood, Adolescent and Women’s Endocrine Health, University of Messina, Viale Gazzi, 98125 Messina, Italy; 3 Interdepartmental Program of Molecular & Clinical Endocrinology and Women’s Endocrine Health, University Hospital, A.O.U. Policlinico G. Martino, Viale Gazzi, 98125 Messina, Italy

**Keywords:** polycystic ovary syndrome, vitamin D, bone mineral density, fracture risk, bone metabolism, insulin resistance, obesity, quality of life

## Abstract

Polycystic ovary syndrome (PCOS) is the most common endocrine disorder among premenopausal women. PCOS may have reproductive, metabolic, cardiovascular, and psychological implications. Vitamin D deficit is often encountered in PCOS women and may contribute to the pathophysiology of this disorder. As of the key role of vitamin D in bone and mineral metabolism, and because the vitamin D status appears to be closely linked with the PCOS manifestations including insulin resistance, obesity, ovulatory and menstrual irregularities, oxidative stress and PTH elevation, hypovitaminosis D may directly and indirectly via the different facets of PCOS impair bone health in these women. Although limited data are available on life-long fracture risk in women with PCOS, the importance of preserving bone health in youth and adults to prevent osteoporosis and related fractures is also recognized in PCOS women. Evidence of the association between vitamin D and the clinical hallmarks of PCOS are summarized and discussed. Vitamin D arises as a cornerstone in women with PCOS and contributes to the pathophysiological link between PCOS and bone metabolism.

## 1. Introduction

Polycystic ovary syndrome (PCOS) is a heterogeneous endocrine disorder affecting up to 20% of premenopausal women, possibly making this syndrome the most common endocrine and metabolic disorder in women of reproductive age. PCOS was first described in 1935 by Stein and Leventhal as the combination of hirsutism (a condition of male pattern terminal hair growth in women), amenorrhea, chronic anovulation and infertility, obesity, and enlarged cystic ovaries [1,2,3]. PCOS is defined in accordance with the Rotterdam criteria (at least two of the following criteria: (1) irregular/no ovulation; (2) clinical/biochemical hyperandrogenemia, and (3) polycystic ovaries [1] or the National Institutes of Health (NIH) criteria (oligo-anovulation and biochemical and/or clinical signs of hyperandrogenism in the absence of other endocrinopathies) or the Androgen Excess and PCOS Society (AE-PCOS) criteria (mandatory presence of hyperandrogenism associated with ovarian dysfunction, defined by oligo-anovulation and/ or ultrasound PCO) [4]. The polycystic appearance of the ovaries frequently found in patients suffering from PCOS is caused by the accumulation of ovarian follicles in different stages of maturation or atresia. The etiology of PCOS remains largely unknown, although it can be considered a complex multigene condition influenced by epigenetic and lifestyle factors [1]. Vitamin D deficiency is defined as 25(OH)D serum levels < 20 ng/mL, while vitamin D insufficiency as serum levels of 21–29 ng/mL [5]. Not adequate vitamin D levels are very common worldwide, not only in the elderly population but also in young people [5]. About 67–85% of women with PCOS show vitamin D deficiency [6]. A poor vitamin D status has been associated with the salient features of PCOS including insulin resistance (IR), ovulatory and menstrual irregularities, decreased pregnancy rate, hirsutism, hyperandrogenism, obesity, and elevated cardiovascular disease [6,7]. Therefore, vitamin D might not only participate in the pathophysiology of PCOS but might also indirectly contribute to lower bone mineral density (BMD) in this particular situation [7]. A poor vitamin D status has been consistently associated with an increased risk of fragility fractures in several clinical settings [8,9,10].

## 2. Vitamin D Metabolism and Vitamin D Role in Female Reproduction 

Vitamin D exists in two forms: ergocalciferol (D2) and cholecalciferol (D3). Vitamin D3 is mainly produced by the skin cells upon exposure to sun rays [11]. A small proportion (about 10–20%) is introduced with food [11]. Then, cholecalciferol is hydroxylated in the liver in 25-hydroxyvitamin D or calcifediol [25(OH)D], the circulating form of the vitamin, whose serum levels are indicative of vitamin D status. In the kidney, 25-hydroxyvitamin D is converted into 1,25-dihydroxy vitamin D or calcitriol [1,25(OH)D] [12]. Calcitriol is the active form of the hormone and its primary function is to regulate calcium/phosphorus metabolism and bone mineralization by osteoclast action [13]. However, vitamin D acts in other tissues such as parathyroid glands, immune cells, pancreas and placenta, uterus, ovaries, and testes via binding to the nuclear vitamin D receptor (VDR) [14,15,16]. VDR is a DNA binding transcription factor, forming a heterodimer with a retinoid X receptor (RXR) [14]. Ramagopalan et al. reported approximately 230 genes in more than 30 different tissues responsive to vitamin D stimulation [16]. Thus, vitamin D has been implicated in a wide range of extra-skeletal effects and diseases, including reproductive dysfunctions and polycystic ovary syndrome (PCOS) [6,17,18,19,20,21,22,23,24,25,26,27,28]. Physiologically, VDR is expressed in reproductive tissues of the cycling mice and pregnant mice, including placenta and decidua [29]. Furthermore, VDR knockout mice have impaired folliculogenesis and hypergonadotropic hypogonadism [30]. In humans, in vitro exposure of ovarian cells to vitamin D increases 3β-HSD mRNA levels and production of progesterone, estrogen, and estrone [13,31,32,33]. Vitamin D stimulates the differentiation and the development of human granulosa cells (GC) and influences follicular maturation by a direct effect on the anti-Mullerian hormone (AMH) gene [13]. AMH, which is considered a marker of the ovarian reserve, is usually produced by the GC of preantral/antral ovarian follicles [34]. Women with PCOS have abnormally increased serum and intrafollicular AMH levels, due to a rise in the number of arrested small antral follicles, besides AMH hypersecretion by the granulosa cells themselves [35]. A study on 54 women undergoing in vitro fertilization described a two-fold increase in AMHR-II expression in GC of women with insufficient follicular fluid 25(OH)D (<30 ng/mL) compared with women having normal follicular fluid vitamin D levels (>30 ng/mL) [31]. It is conceivable that vitamin D alters AMH signaling and steroidogenesis in human cumulus GC [31]. Moreover, a relationship exists between vitamin D and follicle-stimulating hormone (FSH) receptor (FSHR), so vitamin D regulates follicular sensitivity to FSH [5]. Probably, vitamin D regulates both AMHR-II and FSHR gene expression [13]. Although conflicting, these findings suggest that vitamin D influences AMH gene expression and probably, it could neutralize the inhibitory effect of AMH on GC differentiation and follicular growth by inhibiting AMHR-II expression and downstream signaling [33]. A positive correlation between AMH and BMD has been recently found in premenopausal women with suspected ovarian insufficiency [36]. Although this review does not attempt to discuss the pleiotropic effects of vitamin D on female reproduction, these results highlight the crucial role of vitamin D in human biology; it also suggests modifications of bone metabolism in PCOS women via an indirect effect of vitamin D through several hormone changes [15,37]. In other terms, while vitamin D is primarily essential for musculoskeletal health, accumulating data are suggesting that vitamin D may be important for fertility, but also pregnancy outcomes and lactation [38,39]. Thus, by conditioning endocrine PCOS women milieu, vitamin D may secondarily interfere with bone metabolism. 

## 3. Vitamin D and PCOS

**Figure 1:**
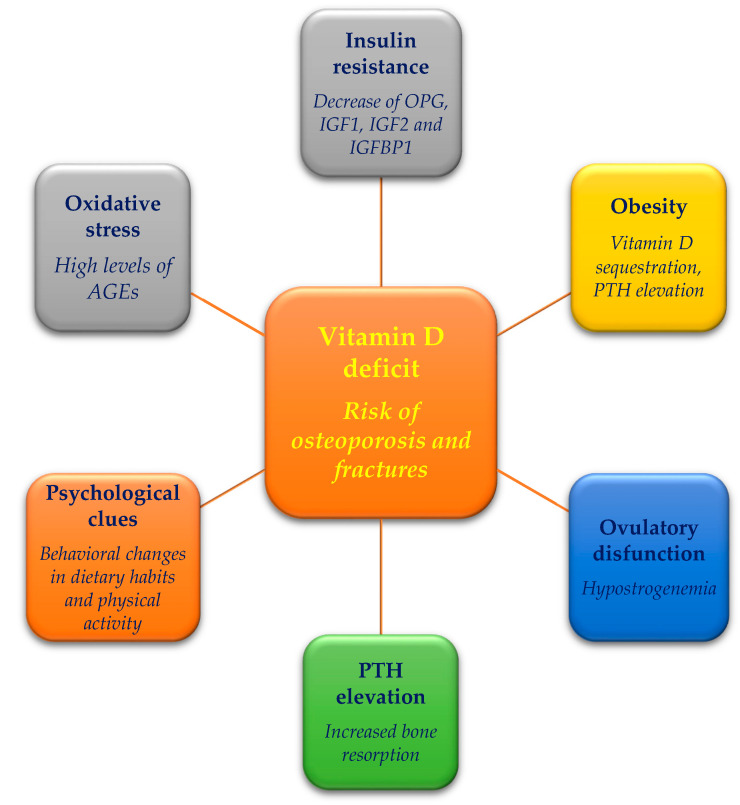
Hypovitaminosis D is associated with several facets of polycystic ovary syndrome, which per se are also related to fracture risk.

As mentioned above, a poor vitamin D status is described in women with PCOS. Several studies reported an involvement of the vitamin D pathway in the development of PCOS and its symptoms, comprising hirsutism, ovulatory dysfunction, IR, and cardiovascular diseases [40,41]. Upon comparing PCOS with non-PCOS women, serum levels of vitamin D levels were reported to be either lower [42,43,44] or statistically similar [45] in PCOS compared with non-PCOS women. Interestingly, interventional studies revealed that vitamin D supplementation might improve menstrual irregularity and follicular development in PCOS women [13]. Additionally, some PCOS clinical features associated with vitamin D, as highlighted in Figure 1, have been previously associated with the risk of osteoporosis and fractures in the general population [46,47]; thus, it is reasonable they could contribute to bone fragility even in PCOS.

### 3.1. VDR Polymorphism 

The binding of the heterodimer VDR-RXR with vitamin-D-responsive elements (VDRE) activates gene transcription, and it has been described that VDR polymorphisms influence the production of its correspondent mRNA, but the exact mechanism is unclear [48]. Several studies described that the gene polymorphism of VDR is associated with a low concentration of 25(OH)D and with the pathogenesis of PCOS metabolic and endocrine features [49]. However, most studies were conducted in Asian countries, and evidence from other geographical areas are needed [48]. VDR polymorphism could regulate the insulin secretor capacity through changes in the quantity of the corresponding gene product [48]. VDR Tru 9I, Bsm1, TaqI, Apa-I, Cdx2, and Fok1 are the most studied polymorphic variants [13,50]. Particularly, Al-Daghri et al. reported a relationship between the VDR Bsm1 variant and the reduced risk of vitamin D deficiency [50]. VDR Apa-I and Bsm1 polymorphisms appear to be associate with an increased risk of PCOS, while TaqI and Cdx2 could be related to severity and susceptibility of PCOS, including strong symptoms such as higher IR, fasting insulin, testosterone levels, body mass index (BMI) and lower vitamin D levels [50]. Furthermore, Cdx2 and FokI variants could be associated with testosterone levels and infertility, respectively [51]. Polymorphisms of vitamin VDR have been also proposed as genetic determinants of bone quality, skeletal geometry, and bone turnover markers [52,53]. Overall, significant correlations between VDR ApaI, VDR FokI, and osteoporosis susceptibility have been found [52,53,54]. A recent meta-analysis has also suggested VDR BsmI genotype is associated with an increased risk of postmenopausal osteoporosis in Caucasians but not in Asians, highlighting possible ethnic differences [54]. Even though results are still conflicting and the molecular mechanisms by which these polymorphisms influence receptor activity remain in part to be investigated, an additional important issue is represented by their potential pharmacogenomic and pharmacogenetic implications [55].

### 3.2. Insulin Resistance and Vitamin D

PCOS is characterized by IR and metabolic dysfunctions and low levels of 25(OH)D levels are negatively associated with IR, BMI, non-HDL cholesterol, blood pressure, leptin levels, androgen levels, but positively with HDL cholesterol [27,56,57,58,59,60,61]. IR is associated with an increased risk for dysglycemia, type 2 diabetes mellitus, metabolic syndrome, and cardiovascular disease [14,62]. Women with PCOS and vitamin D deficiency have a greater prevalence of impaired glucose tolerance compared with PCOS women with no vitamin D deficiency [6]. Vitamin D appears to regulate the glyco-insulin homeostasis via different mechanisms: (1) stimulating insulin release through the expression of VDR in the pancreatic β-cells; (2) increasing responsiveness of GLUT (glucose transport) to insulin through the binding of the 1,25(OH)D–VDR complex to the VDRE of the insulin receptor at the tissue level; (3) suppressing of the release of proinflammatory cytokines that are believed to mediate IR; (4) regulating of intra and extracellular calcium levels, which are essential for insulin-mediated actions [62,63,64,65]. At the bone tissue level, insulin physiologically increases osteoblast proliferation and collagen formation and inhibits parathyroid hormone (PTH) action; conversely, the state of hyperinsulinism leads to a decrease of osteoprotegerin (OPG) as well as insulin-like growth factors 1 and 2 and insulin-like growth factor-binding protein 1, increasing bone reabsorption [66,67]. The decrease in OPG/RANKL ratio can result in an increased expression of Tcirg1 in osteoclast, which encodes for a proton pump and contributes to the acidification of the resorption lacunae. The acidic pH can decarboxylate and activate osteocalcin. The undercarboxylated form of osteocalcin, on the other hand, has been shown to facilitate pancreatic β-cell proliferation and insulin secretion [68]. The treatment with both vitamin D and calcium appears to reduce IR and serum androgen levels in vitamin D-deficient PCOS women with consequent improvement of hirsutism and menses regularity [69,70,71]. In a recent review, supplementation of vitamin D < 4000 IU/d or administration of vitamin D as a co-supplement improved insulin sensitivity reducing the fasting glucose concentration (about 6.3% with supplementation with vitamin D and other micronutrients), the mean fasting insulin levels (about 22% in some trials) and HOMA-IR [72]. However, Menichini and Facchinetti, after reviewing the effects of vitamin D supplementation in women with PCOS, showed better outcomes after the supplementation of high dose (4000 IU), compared with low-dose (1000 IU) of vitamin D or placebo, for a period of at least 12 weeks [73]. In addition, a recent meta-analysis, including 10 randomized controlled trials, has shown a significant reduction of fasting glucose levels but no significant effect on fasting insulin concentration and HOMA-IR, in vitamin D-deficient PCOS women [74]. Overall, vitamin D supplementation appears to have beneficial effects on IR and dysglycemia and could as well modulate bone metabolism [66,67,75,76,77].

### 3.3. Hyperandrogenemia and Vitamin D

The low serum levels of FSH and the increased serum levels of luteinizing hormone characterizing PCOS, stimulate the androgen synthesis and the subsequent development of IR [31]. IR leads to the enhanced ovarian secretion of androgens and consequently reduction of sex hormone-binding globulin (SHBG) production [61], low SHBG concentrations determining elevated free serum testosterone levels [78]. Furthermore, hyperandrogenemia is a principal causal factor of the metabolic dysfunctions observed in PCOS [79]. Indeed, hyperandrogenemia is often accountable for impaired insulin sensitivity and it influences the distribution of adipose tissue with the development of insulin-signaling abnormalities and IR, abnormal visceral adiposity, and adipose tissue dysfunction, thus determining a vicious circle [79]. Androgen receptors are expressed by osteoblasts, osteoclasts, and osteocytes in both men and women, suggesting a direct action of androgen on these bone cells [80]. However, androgens could affect bone metabolism indirectly, e.g., via the inhibition of bone resorption by downregulating interleukin-6 (IL-6) and prostaglandin E (PGE) synthesis, or via the inhibition of PTH release by increasing intestinal calcium absorption and preventing its excretion and by increasing vitamin D3 production. Androgen receptors in bone are upregulated not only by androgens, but also by estrogens, glucocorticoids, and 1,25(OH)D; however, serum levels of estrogens and 1,25(OH)D are reduced in patients affected form PCOS [81]. This aspect of PCOS may adversely affect the regulation of BMD by androgens, although BMD is almost always positively associated with testosterone levels. It is also known that vitamin D deficiency is associated with abnormalities in serum DHEAS, testosterone, SHBG, and free androgen index (FAI) [6]. For instance, some studies on PCOS women have reported inverse associations between serum 25(OH)D levels and testosterone, DHEAS and FAI and SHBG [44,58,82], and lower 25(OH)D levels have been detected in hirsute PCOS women in comparison with BMI-matched controls [15]. Vitamin D supplementation has significantly decreased serum total testosterone, serum free-testosterone, and DHEAS, even if it has been ineffective in improving other androgenic markers, such as SHBG [83,84,85]. Vitamin D supplementation has been shown to improve features of PCOS-related metabolic syndrome [86,87]. Menichini and Facchinetti reported that vitamin D supplementation at high doses (4000 IU/d) for at least 12 weeks, can improve serum levels of SHBG, FAI, and total testosterone [73]. In a randomized clinical study, vitamin D supplementation at 50,000 IU/week for 12 weeks in 30 over-weight PCOS-women decreased hirsutism score, FAI, and increased SHBG and 25(OH)D levels, with significant changes in ovaries ultrasonography and menstrual cycle regularity [88]. However, randomized placebo-controlled trials are needed to confirm the beneficial effect of vitamin D on hyperandrogenemia and to determine the dose and the duration of vitamin D treatment required to improve the androgenic profile [83].

### 3.4. Oxidative Stress and Vitamin D 

A relationship exists between vitamin D deficiency and oxidative stress in the pathogenesis of PCOS [89]. Merhi and colleagues reported high levels of advanced glycation end-products (AGEs), produced endogenously by a combination of oxidation and glycation or absorbed exogenously from modern heat-processed diets in women with PCOS [90]. Both AGEs and their anti-inflammatory soluble receptors, sRAGE, have a role in the metabolic and reproductive features of PCOS [90]. AGEs accumulate in granulosa and techa cell layers of PCOS women with consequent worsening of follicular growth [91,92]. sRAGE levels in follicular fluid are reduced in PCOS women [93]. It has been observed that vitamin D supplementation in PCOS women may improve the steroidogenesis and enzymatic antioxidant activity in the human GC [94] and it may attenuate the actions of AGEs [95,96]. AGEs play a role in age-related bone loss [97,98]. AGEs provoke bone cell impairment and alter bone biomechanical properties. Pentosidine (PENT), a well-characterized AGE, is even considered a predictor of bone fracture [97]. It has been reported AGEs interfere with osteoblast maturation, leading to morphological cell modifications, function failure, and inhibition of the calcification process. In vitro, coadministration of vitamin D and vitamin K2 prevented the AGEs related inhibition of ALP secretion and up-regulated collagen gene expression. In contrast, in the absence of vitamin D and vitamin K2, intracellular collagen and osteocalcin levels were decreased and the RANKL/OPG ratio was increased after PENT exposure [99]. These pieces of evidence suggest a favorable vitamin D status may promote bone strength also via contrasting AGEs impact on bone metabolism. 

### 3.5. Parathyroid Hormone (PTH) and Vitamin D in PCOS

Vitamin D and calcium metabolism are linked with obesity in PCOS women [41,45]. Probably, the obesity-related deficiency of vitamin D is attributable to a more sedentary lifestyle that leads to less sun exposure and consequently reduced cutaneous vitamin D production and/or vitamin D sequestration in subcutaneous adipose tissue [100]. Due to vitamin D deficiency, obese individuals have higher serum PTH levels [101]. Furthermore, PTH itself could favor obesity by increasing intracellular calcium concentrations, which in turn seems to promote triglycerides accumulation and inhibit lipolysis [102]. PTH concentration is increased in PCOS women compared with BMI-matched controls and in obese PCOS women compared with normal-weight PCOS women [45,103]. Studies have also revealed a direct correlation between PTH concentration and serum levels of testosterone, independently of BMI [45], and an inverse association between vitamin D concentration and androgen levels [104]. Adrenal androgen excess in PCOS is associated with increased inactivation of cortisol, which in turn leads to elevated levels of TNFα and IL-1β potentially resulting in chronic inflammatory bone disease and bone loss [105]. PTH is also supposed to directly stimulate the adrenal cortex through interaction with PTH receptor 1 [106]. It is possible there is a direct relationship between PTH and PCOS and that the beneficial effects of vitamin D supplementation on hyperandrogenemia are mediated, at least in part, by the vitamin D action on insulin sensitivity [45]. Overall, the increased circulating PTH levels, because of low 25(OH)D levels in PCOS, may drive bone loss by promoting bone resorption [105,107].

### 3.6. Fracture Risk in PCOS 

Few studies have addressed bone health and fracture risk in women with PCOS (Table S1). The largest register-based cohort study considered a total of 19,199 women (age 12–60 years) in Denmark and found a decreased fracture risk [adjusted hazard ratio (aHR) = 0.76, 95% CI = 0.71–0.80 for all fractures; 0.82, 95% CI = 0.74–0.92 for major osteoporotic fractures] [108]. Conversely, an observational population-based study of 11,106 women (age 15–80 years) with PCOS in Taiwan reported an increased incidence of any fractures in comparison with non-PCOS women [aHR= 1.23, 95% CI = 1.13–1.33]; particularly, the aHR of osteoporotic fractures was 1.33, 95% IC = 1.15–1.54; that of spine fractures was 1.36, 95% IC = 1.11–1.66 and that of forearm fractures was 1.39, 95% IC = 1.07–1.80 [109]. Of note, the Denmark study [108] included women with hirsutism in addition to women with PCOS, which may have contributed to the heterogeneity of the study findings. Also, differences between European and Asian patients with PCOS may exist, as Chinese women with PCOS have a higher prevalence of polycystic ovarian morphology, less severe hyperandrogenism, and lower prevalence of impaired glucose tolerance and IR compared with Caucasian women [109]. However, these two studies did not analyze the potential role of confounders including body weight or BMI, lifestyle factors, and physical activity that could be involved in the peak of bone mass and the pathophysiology of bone loss later in life [110]. Yang et al reported PCOS women had a significantly higher incidence of osteoporotic fractures, spine fractures, and forearm fractures in comparison with matched healthy controls, but the similar incidence in femur or hip, humerus, wrist, and non-osteoporotic fractures [108]. Inconsistent results were reported also for BMD measurements in PCOS women. BMD is a major determinant of bone strength, but fragility fractures can occur also in women with normal or slightly reduced BMD as observed in the general population [109]. In PCOS women, some studies reported lower BMD values [111,112,113,114,115], while others reported no difference in BMD in comparison with healthy controls [116,117,118,119,120,121,122,123,124,125,126,127,128], probably due to differences in selection criteria and age of participants. A systematic review and meta-analysis showed that, in comparison with controls, women with PCOS and BMI < 27 kg/m², but not women with PCOS with BMI ≥ 27 kg/m², have decreased spinal and femur BMD, suggesting a role for adiposity in contributing to bone parameters [66]. Women with PCOS show also impaired bone metabolism as highlighted by studies that analyzed surrogate markers of bone turnover; namely carboxy-terminal collagen type 1 telopeptide, which is a bone resorption marker, and procollagen type 1 N-terminal propeptide and osteocalcin, which are bone formation markers [109,117,121,129,130,131,132,133]. Overall, PCOS women were reported to have decreased serum levels of bone formation markers that could negatively impact BMD and ultimately enhance the risk of fractures [60].

### 3.7. Vitamin D and Clinical Psychological Features in PCOS

PCOS women have been documented to be at risk for psychological distress. In a cross-sectional analysis based on the Australian Longitudinal Study of Women’s Health (ALWSH), comparing women with (*n* = 478) and without (*n* = 8134) a self-reported diagnosis of PCOS, women with PCOS showed a higher prevalence of depression, anxiety symptoms and greater score for perceived stress [132]. A number of studies have associated low 25(OH)D levels with poor mental health, depression, and anxiety [133,134,135,136,137,138,139]. Using the validated Hospital Anxiety and Depression Score questionnaire, Moran et al reported a positive association between depression and weight, BMI, waist circumference, and highly sensitive C-reactive protein (hsCRP) in women with PCOS [140]. Additionally, in a sensitivity analysis including Caucasian women who had no history of psychiatric illness or current use of vitamin supplements or psychiatric medication, a significant negative association between 25(OH)D and depression score was observed. At a multiple regression analysis, the 25(OH)D concentration was the only significant predictor of CRP and depression, so that for each 1.0 nmol/L decrease in serum levels of 25(OH)D, there was an increase by 0.041 mg/L in serum levels of CRP and an increase by 0.063 points in depression score [133]. The correlation between 25(OH)D and depression in PCOS reflects what is observed in the general population. It may depend on behavioral modifications, including changes in dietary habits and physical activity leading to weight gain or changes in sun exposure. Depression may seriously affect bone health and increase the risk of fragility fractures [141,142]. High levels of serum PTH, also due to poor vitamin D status, have been observed in major depression, with these high levels contributing to bone loss [143]. However, PCOS was not per se an independent predictor of depression, but it was the only independent predictor of anxiety [140]. Anxiety has been recently proven to predict low BMD and increased fracture risk in middle-aged women [142,144]. Both depression and anxiety have been related to low vitamin D concentrations [145,146]. In this context, quality of life, which is also influenced by psychological features, is lower in women with poor 25(OH)D, underlining the role of vitamin D as a marker of both physical and mental health [147,148]. 

## 4. Conclusions

Vitamin D is a hormone with pleiotropic and multiple functions. Beyond bone metabolism, it influences the reproductive axis and plays a role in the pathogenesis of PCOS. Indeed: (1) PCOS women showed a relative vitamin D deficiency (in 67–85% of cases); (2) Several VDR polymorphisms are linked with PCOS and its severity phenotypes; (3) An association exists between low vitamin D levels and each of obesity, hyperandrogenism, IR, and other metabolic dysfunctions that are PCOS-related; (4) Bone health can be influenced by several facets of PCOS possibly resulting in an increased risk of fracture over time; (5) Hypovitaminosis D is directly and indirectly linked to poor bone health in PCOS; (6) Supplementation of vitamin D-deficient PCOS women with vitamin D may improve different aspects of this disorder including menstrual regularity, fertility, BMI, lipidic profile, IR, cardiovascular risk and probably bone health. For all its beneficial properties, cheapness, and safety, vitamin D supplementation could be inserted into the therapeutic options of PCOS women, in addition to insulin-sensitizing agents and antioxidants, regardless of BMI. However, further trials are needed to clarify the role and all the possible effects of vitamin D on PCOS parameters, comprising bone health and the life-long fracture risk.

## Supplementary Materials

The following are available online at https://www.mdpi.com/2218-1989/11/2/116/s1, Table S1: Main studies focused on bone health in polycystic ovary syndrome.
