# Supplementary material for: Vitamin D, Bone Metabolism, and Fracture Risk in Polycystic Ovary Syndrome
Source: Metabolites. 2021 Feb 18;11(2):116. doi: 10.3390/metabo11020116 (PMC7922814; doi:10.3390/metabo11020116)
Supplement: Supplementary file 1 [file metabolites-11-00116-s001.pdf]

Table 1. Main studies focused on bone health in polycystic ovary syndrome.

Study	Study design	Participants	Outcomes	Main findings
**Adami, S. et al, 1998** *(Ref. 117)*	Cross- sectional study	51 women with PCOS, 24 women with idiopathic hirsutism, 26 women with hypothalamic amenorrhoea, 35 healthy women	Lumbar spine BMD<br>Left femoral neck BMD<br>Bone metabolism markers	Lumbar spine and femoral BMD were significantly decreased and bone markers (serum osteocalcin, and urinary excretion of free deoxypyridinoline, cross-linked N-telopeptide and hydroxyproline) significantly increased in patients with hypothalamic amenorrhoea, when compared to control subjects and the other two patient groups. In the sub-group of PCOS patients with amenorrhoea, spine and femoral neck BMD was significantly lower than in patients with idiopathic hirsutism and the non-amenorrheic PCOS patients.
**Good, C. et al, 1999** (*Ref. 119*)	Cross-sectional study	20 PCOS women<br>10 healthy controls	Total body BMD	Androgen levels were significantly elevated in the lean PCOS women compared with the controls. There was no statistically significant difference in total body BMD between the two groups. Evaluation of upper body BMD showed a significant correlation between testosterone levels and BMD.
**Yüksel, O. et al., 2001** *(Ref. 111)*	Cross- sectional study	28 PCOS women, 11 amenorrheic women without PCOS, 15 healthy women	Lumbar spine BMD<br>Left femoral neck BMD	BMD at the lumbar spine (0.82±0.20 g/cm²) and at femoral neck (0.95±0.02 g/cm²) in the PCOS group was significantly higher than in the amenorrheic control group (0.72±0.02 and 0.88±0.02; p< 0.05 and p<0.001, respectively), and lower than in the healthy control group (0.92±0.02 and 1.04 ±0.02, respectively; p<0.05).
**Kirchengast, S. et al, 2001** *(Ref. 116)*	Cross- sectional study	10 PCOS women, 10 healthy women	Total body BMD	BMD was almost identical in PCOS and controls.
**Noyan, V. et al, 2004** (*Ref. 118*)	Cross- sectional study	29 PCOS women, 17 healthy controls	Total body BMD<br>Lumbar spine BMD<br>Femoral neck BMD	BMD measurements did not differ between the groups, but fasting insulin was significantly higher and fasting glucose/insulin ratio was significantly lower in the PCOS group compared to the controls (p=0.021 and 0.008, respectively) and there were significant correlations between fasting insulin and total BMD (r=0.424, p<0.05) and fasting glucose/insulin ratio and L2–L4 BMD (r= -0.401, p< 0.05) after controlling for age and BMI.
**Glintborg, D., et al, 2008** *(Ref. 120)*	Randomized clinical study	30 PCOS women, 14 healthy controls	Lumbar spine BMD and femoral BMD<br>Bone metabolic markers Vitamin D	Patients with PCOS had significantly higher levels of C-terminal telopeptide (CTX) than controls, whereas no differences were measured in ALP, 25(OH)D, PTH, body composition, or BMD. In patients with PCOS, total hip BMD significantly correlated with BMI (r=0.41; p=0.02), trunk fat mass (r =0.41; p=0.02), trunk lean body mass (r=0.46; p=0.01), and lower-extremity lean body mass (r=0.43; p=0.02).
**Carmina, E. et al, 2009** *(Ref. 127)*	Cross- sectional study	95 PCOS women, 90 healthy women	Total body BMD	BMD was almost identical in PCOS and controls.

**Kassanos, D., et al, 2010** (*Ref. 126)*	Cross- sectional study	30 PCOS women, 15 healthy women	Peripheral quantitative computed tomography (pQCT) of bone	Using pQCT, the following parameters were measured: volumetric cortical density (CBD) and volumetric trabecular density (TBD) BMD, total bone cross-sectional area (ToA), cortical area (CoA), cortical thickness (CRT-THK-C) and finally the strength-strain index (SSI). The geometrical parameters (CoA, ToA, CRT-THK-C), the SSI as well as the TBD were increased in the PCOS women; however, these differences did not achieve statistical significance between lean PCOS women, obese PCOS women, and controls.
**Diamanti-Kandarakis, E. et al, 2011** *(Ref. n. 129)*	Cross-sectional study	50 PCOS women, 47 matched controls	Bone metabolic markers	Osteocalcin (4.30±1.74 vs. 6.20±1.78 ng/ml, $p<0.0005$) values were significantly lower, whereas carboxylated osteocalcin (37.93±6.87 vs. 9.64±8.21 ng/ml, $p<0.0005$) and receptor activator for nuclear factor-kB ligand (0.54±0.26 vs. 0.16±0.15 pmol/l, $p<0.0005$) values were significantly higher in PCOS subjects compared to the control group, independently of obesity. A significant association was disclosed between osteocalcin and carboxylated osteocalcin with androgens, insulin resistance, advanced glycated end products (AGEs), and ovarian morphology.
**Schmidt, J. et al, 2012** *(Ref. 123)*	Prospective study (follow up from 1987 to 2008)	25 PCOS women 68 age-matched controls	Total body BMD Lumbar spine BMD Femoral BMD Radius BMD Fractures	At follow-up, the postmenopausal women with PCOS maintained a higher free androgen index (FAI), but had similar body fat, lean mass and BMD compared with controls. The hip circumference increased only in women with PCOS ($p< 0.01$), during follow-up. The fracture incidence was similar to that of controls (56% vs. 41%, ns). In the controls, total BMD was positively correlated with estradiol ($r =0.322$, $p< 0.01$) and FAI ($r=0.307$, $p<0.05$) and negatively correlated with sex hormone-binding globulin (SHBG) ($r= −0.429$, $p< 0001$), but not in the women with PCOS.
**Pepene, C.E., et al, 2013** *(Ref .131)*	Cross-sectional study	52 PCOS women 26 matched controls	Bone metabolic markers	BMI-stratified multivariate analysis revealed significantly higher osteocalcin levels in PCOS *vs* controls in lean ($p = 0.001$) but not overweight and obese study participants. A positive correlation between osteocalcin and TT ($p = 0.018$) and serum insulin ($p=0.036$), respectively, was found to be confined to the lean analysis subgroup. β-CrossLaps were not significantly different in PCOS women in comparison with controls.
**Katulski, K. et al, 2014** *(Ref. 112)*	Cross-sectional study	69 PCOS women 30 age-matched controls	Lumbar spine BMD	PCOS women had lower BMD values as compared to the controls (1.057±0.1260 vs. 1.210±0.1805 g/cm², $p<0.0002$). In the analysis of PCOS patients according to BMI, only in the subgroup of the normal weight PCOS showed significantly lower BMD in comparison to controls ($p=0.0049$). In patients with PCOS, BMD was positively associated with insulin concentration and HOMA–IR.
**Berberoglu, M. et al, 2014** **(***Ref. 125)*	Cross-sectional study	42 PCOS women 20 healthy controls	Plasma GDF-9 and GDF-15 concentrations, bone turnover markers and BMD	No significant differences were observed in bone turnover markers, BMD measurements and plasma levels of growth and differentiation factor (GDF)-9 and GDF-15, in subjects with PCOS compared with controls.
**Attlee, A. et al, 2014** *(Ref. 115)*	Cross-sectional study	40 PCOS women 10 healthy controls	BMD	The participants with PCOS had lower BMD than those without it, whereas the difference was statistically non-significant, as measured using a body composition analyser.
**Rubin, K. H. et al., 2016** *(Ref. 108)*	Observational Danish register-based cohort study (1995–2012)	19.199 PCOS, 57.483 controls, 12-60 (mean 30.6) yrs	All fractures Major osteoporotic fractures*	Adjusted OR 0.76 (95% CI, 0.71 to 0.80) for all fractures. Adjusted OR 0.82 (95% CI, 0.74 to 0.92) for major osteoporotic fractures.
**Gao, S. et al, 2016** *(Ref. 121)*	Cross-sectional study	52 PCOS women, 39 controls	Irisin Body composition BMD	Serum irisin level of PCOS did not show a significant difference compared with control subjects although it was decreased. Levels of adiponectin in PCOS patients were significantly decreased compared with controls. No differences were found in BMD between control and PCOS women ($p >0.05$). However, PCOS women showed significantly higher levels of head fat%, android fat%/gynoid fat% (A/G), fat mass/height², %fat trunk/%fat legs, total lean mass, trunk lean mass, subtotal lean mass, lean mass/height², and appendicular lean mass/height², and lower levels of trunk/limb fat mass ratio than controls in body composition. Partial correlations analysis indicated a positive correlation between serum irisin levels and BMD in the control group and a negative correlation in the PCOS group, after BMI and age adjusted.

**Albaik et al, 2016** *(Ref. 124)*	Cross-sectional study	36 PCOS women<br><br>36 healthy matched controls	Lumbar spine BMD<br>Femoral BMD<br>Vitamin D	Women with or without PCOS did not show any significant differences in BMD values, at both lumbar spine (L1-L4) and femoral neck. Serum 25(OH)D and PTH did not show significant differences between PCOS and control group or between lean and obese subgroups.
**Kalyan, S. et al, 2017** *(Ref. 113)*	Cross-sectional study	22 women with PCOS,<br><br>39 controls	Total hip BMD<br><br>Radius strength-strain index [SSI] by pQCT	A diagnosis of PCOS negatively predicted (beta= −0.251, p=0.022) hip BMD in a regression model including weight. In PCOS women, inflammation (C-reactive protein/albumin) was inversely associated with radial SSI (R^2 =0.25, p=0.018).
**Karadağ C. et al, 2017** *(Ref. 114)*	Cross-sectional study	103 PCOS women<br><br>60 healty controls	Lumbar spine BMD<br>Femoral BMD	Lumbar BMD and femoral neck BMD were significantly lower in PCOS group than controls (p<0.01). In PCOS group, hyperandrogenemic women had higher BMD values than normoandrogenemic PCOS patients (p<0.01). In PCOS group, lumbar BMD was significantly correlated with HOMA-IR (r=0.617; p<0.01), MATSUDA ISI (r= -0.665; p<0.01), serum E2 (r=0.488; p<0.01), total testosterone (r=0.436; p<0.01), and androstenedione (r = 0.337; p<0.01) levels. Similar correlations observed for femoral BMD.
**Lingaiah S et al, 2017** *(Ref. 130)*	Cross-sectional study	298 PCOS women<br><br>194 healthy controls	Bone turn-over markers<br>Vitamin D	Serum levels of amino-terminal propeptide of type 1 procollagen (P1NP) and osteocalcin were decreased in women with PCOS compared with controls, whereas no significant differences were found in CTX and 25(OH)D levels. Age-stratified analyses suggested that P1NP and osteocalcin levels were decreased only in the younger age group (≤30 years) women with PCOS compared with controls. The formation markers and resorption marker decreased with age in both study groups. No significant differences were found in 25(OH)D levels.
**Yang, H.-Y. et al., 2018** *(Ref. 109)*	Observational Chinese population-based cohort study (2000-2012)	11.106 PCOS,<br>44.424 non-PCOS<br><br>15-80 yrs	Any fractures<br>Osteoporotic fractures<br>Spine fractures<br>Forearm fractures	Adjusted HR 1.23 (95% CI, 1.13 to 1.33) for any fractures.<br>Adjusted HR 1.33 (95% CI, 1.15 to 1.54) for osteoporotic fractures.<br>Adjusted HR 1.36 (95% CI, 1.11 to 1.66) for vertebral fractures.<br>Adjusted HR 1.39 (95% CI, 1.07 to 1.80) for forearm fractures.
**McBreairty, L. E. et al, 2018** *(Ref. 122)*	Controlled clinical study	60 PCOS women,<br><br>60 healthy controls	Total body BMD<br>Lumbar spine BMD<br>Femoral BMD<br>hip geometry	Measures of hip geometry, including cross-sectional area, cross-sectional moment of inertia, subperiosteal width (SPW), and section modulus, were similar between groups after correcting for BMI (all p>0.05) with intertrochanter SPW significantly lower in women with PCOS (p<0.05). BMI-corrected whole body BMD, as well as the lumbar spine and regions of proximal femur were also comparable between groups. In women with PCOS, BMI-corrected correlations were found between insulin and femoral shaft SPW (r=0.322, p<0.05), glucose and femoral neck (r=0.301, p<0.05), and trochanter BMD (0.348, p<0.05), as well as between testosterone and femoral neck BMD (0.376, p<0.05) and narrow neck cross-sectional area (0.306, p<0.05).

Legend: PCOS = Polycystic ovary syndrome; BMD = Bone Mineral Density; BMI = body mass index; **Spine, humerus, and forearm fractures.*
